# Frontal Bone Insufficiency in *Gsk3β* Mutant Mice

**DOI:** 10.1371/journal.pone.0149604

**Published:** 2016-02-17

**Authors:** Heather Szabo-Rogers, Wardati Yakob, Karen J. Liu

**Affiliations:** Craniofacial Development and Stem Cell Biology, Floor 27, Tower Wing, Guy’s Campus, King’s College London, London, United Kingdom SE1 9RT; University of Massachusetts Medical, UNITED STATES

## Abstract

The development of the mammalian skull is a complex process that requires multiple tissue interactions and a balance of growth and differentiation. Disrupting this balance can lead to changes in the shape and size of skull bones, which can have serious clinical implications. For example, insufficient ossification of the bony elements leads to enlarged anterior fontanelles and reduced mechanical protection of the brain. In this report, we find that loss of *Gsk3β* leads to a fully penetrant reduction of frontal bone size and subsequent enlarged frontal fontanelle. In the absence of *Gsk3β* the frontal bone primordium undergoes increased cell death and reduced proliferation with a concomitant increase in *Fgfr2-IIIc* and *Twist1* expression. This leads to a smaller condensation and premature differentiation. This phenotype appears to be Wnt-independent and is not rescued by decreasing the genetic dose of *β-catenin/Ctnnb1*. Taken together, our work defines a novel role for *Gsk3β* in skull development.

## Introduction

The frontal bones develop from neural crest derived mesenchymal cells that initially condense in a position dorsal to the developing eyes. Following the initial condensation, the frontal bones grow by expansion and dorsal migration of the initial cellular condensation [1,2]. These condensations subsequently undergo intramembranous ossification. A number of molecular signals have been implicated in skull growth and patterning, including bone morphogenetic protein (BMP), fibroblast growth factor (FGF), Hedgehog (Hh) and Wnt pathways [3]. In particular, there appears to be a key role for Wnt/β-catenin signaling in the development and ossification of the skull bones [4–7], as well as a requirement for the Wnt inhibitor *Axin2* [4,6–10]. However, to date, there have been no reports of a role for glycogen synthase kinase-3 (*Gsk3*), a key Wnt effector, in the initiation of the frontal bone condensation.

GSK3 is a promiscuous serine/threonine kinase initially identified for its role in glycogen metabolism. In mammals, *Gsk3* is encoded by two paralogs, *Gsk3α* and *Gsk3β*; each has a unique developmental expression pattern in the skull [11]. During embryogenesis, GSK3 is thought to function primarily in the Wnt signaling pathway, where β-catenin degradation is controlled by GSK3 dependent phosphorylation. However, GSK3 has many potential substrates, including GLI proteins, which transduce Hedgehog signaling ([12–14], the insulin receptor IRS-1 and TWIST1 [15,16]. Given the broad function of the two GSK3 proteins, it is surprising that single gene knockouts have minimal phenotypes: the *Gsk3α* knockout animals are viable [11,17], while *Gsk3β* knock out animals survive to birth and die due to cleft palate [18–21].

In humans, malformations of the craniofacial skeleton are among the most common congenital anomalies seen in live births. These anomalies include defects in osteogenesis in the skull vault. Premature closure of the cranial sutures fuses the bones of the skull vault together and results in craniosynostosis, while prolonged patency of the sutures results in enlarged fontanelles and unossified regions between the bones in the skull vault. Both of these abnormalities may be caused by disruptions in the intramembranous ossification programme and indeed, key pathways such as BMP and FGF signaling are implicated in disease pathology [22,23]. Several important genes, *Msx2* and *Twist1*, have been directly linked to enlarged fontanelles, but clearly these cannot be the only candidates [22].

Here, we describe the requirements for *Gsk3β* during development of the neural crest derived frontal bones. In the absence of *Gsk3β*, the frontal bone primordia are small, leading to enlarged fontanelles. In *Gsk3β* mutants, we observe reduced proliferation and increased apoptosis at E13.5. Concurrently, the expression of key differentiation markers *Fgfr2-IIIc* and *Twist1* are increased. These data imply premature differentiation of the frontal bones leading to a depletion of the endogenous store of differentiating osteoblasts. Thus, GSK3β appears to be a key regulator of the balance between growth and differentiation in the embryonic skull.

## Materials and Methods

### Animals

All animals were housed in the New Hunt’s House Biological Services Unit at King’s College London. There are three null alleles of *Gsk3β* [11,18,20]. We have previously shown that these three lines are allelic and phenotypically identical [11,18]. In brief, all three alleles lead to a loss of function protein. The original allele of *Gsk3β* (*Gsk3β*^*tm1Jrw*^) is a conventional null with a neomycin cassette replacing the ATP-binding loop [20]. Although this allele was initially reported to be lethal in mid-gestation, further analysis by us and others demonstrate that these animals undergo late gestational or perinatal death [11,18,19,21]. This is confirmed by perinatal lethality in a second null allele (*Gsk3β*^*tm1Dgen*^) which has a *lacZ* gene replacing the first exon of the protein [11]. Finally, the *GSK3β*^*tm1Grc*^ allele has a protein destabilization domain fused to the 3’ end of the protein which renders it phenotypically null until restored by administration of rapamycin or rapamycin analogues [18,24]. We have verified via western blot that no GSK3β protein is detected in any of the alleles and therefore, all three lines have been used interchangeably in these analyses and, for simplicity, are referred to as *Gsk3β*^*-/-*^. All conclusions from *Gsk3β* mutants were based on at least three animals of the same genotype, with comparison to littermate controls. For neural crest lineage tracing, the *Wnt-1*::*cre* driver and *R26R*^*lacZ*^ reporter lines were used as previously reported [25–27]. In order to generate heterozygous deletions of *β-catenin*, *β-catenin*^*fl/fl*^ mice were crossed to *β-actin*::*cre* driver mice[28,29].

### Mouse husbandry

Gestation dates were determined by observation of a vaginal plug, which was designated as embryonic day 0.5 (E0.5). On the indicated days, the pregnant dams were euthanized by CO2 inhalation, or cervical dislocation and the embryos were then collected by caesarian section. All conclusions were based on a minimum of 3 animals per genotype and the phenotypes that we are reporting here are consistent amongst all of the animals that we analyzed. All animal work was approved by the Ethical Review Board at King’s College London and performed in accordance with United Kingdom Home Office Licenses 70/6607 and 70/7441.

### mRNA *in situ* hybridization

Embryos were fixed overnight at 4°C in 4% paraformaldehyde in phosphate buffered saline. Embryos were processed for paraffin embedding and sectioning according to standard protocols, and 10 micron thick sections were mounted on 3-triethoxysilylpropylamine (TESPA) treated Superfrost slides. mRNA *in situ* hybridization was performed according to standard protocols and revealed with BM Purple [27]. For each probe, control and mutant sections were treated and developed together, and the conclusions were based on at least 3 animals/genotype per gene. The following probes were used: *alkaline phosphatase*, *Cbfa1*, *Osx1* [30], *Fgfr2-IIIc* [31], *Twist1* [32], *Msx1* and *Msx2* [33], *Gsk3α* clone (accession #BC111032, Open Biosystems clone ID 5369444) and *Gsk3β* (accession #BC006936, Open Biosystems clone ID 2648507).

### Skeletal staining

Whole mount E18.5 bone and cartilage preparations were performed as previously described [18]. E15.5 skull preparations were fixed overnight in 4% PFA to maintain tissue integrity. These skulls were subsequently stained with alizarin red and cleared in 1% potassium hydroxide. Histological identification of bone and cartilage on sections was performed using traditional picrosirius red/alcian blue staining protocols [34].

### Wholemount *lacZ* staining

In the lineage tracing experiments, β-galactosidase activity was visualized by X-gal staining as previously described [27]. Cre negative littermates were used to ensure specificity of staining.

### Cell death and cell proliferation

Cell death was examined by TUNEL staining on slides using the ApopTag Peroxidase kit (Millipore). Mitotic cells were identified by antibody staining for phospho-histone H3 (PHH3, Cell Signaling) using a standard citrate buffer antigen retrieval and detection with a peroxidase conjugated secondary antibody. To track DNA synthesis, 10 mg/kg bromo-deoxyuridine (BrdU) was administered to pregnant dams by intraperitoneal injection two hours prior to harvesting. Briefly, sections were pre-treated with proteinase K, exonuclease III and DPN1, and BrdU was detected with the anti-BrdU antibody (RPN202; GE Healthcare) [35]. In each case, at least 1 section from 2 animals was counted per genotype. We counted the positive cells in each frontal bone primordia, and because it consists of ordinal data, it cannot be averaged. We found that the data fell naturally into two categories. With the PHH3 data, there were 8 sections that were below 6 positive cells, while the remaining sections were greater than 6 cells. For the TUNEL data, no wildtype section had greater than 3 apoptotic cells, therefore we analyzed the data using these categories.

### Western Blotting

Tissues were lysed in RIPA, and proteins were separated on a 4–12% NuPAGE Bis-Tris gel with MOPS running buffer (Invitrogen). They were transferred to PVDF membranes and incubated with the activated β-catenin (CTNNB1) antibody (Millipore 8E7), followed by GSK3α /β antibody (Santa Cruz 0011-A) and HSP-90 (Santa Cruz). The signal was detected using the Millipore Immobilon Western Chemiluminescent HRP substrate detected with X-ray film.

## Results

### Loss of *Gsk3β* results in congenital craniofacial anomalies

**Fig 1:**
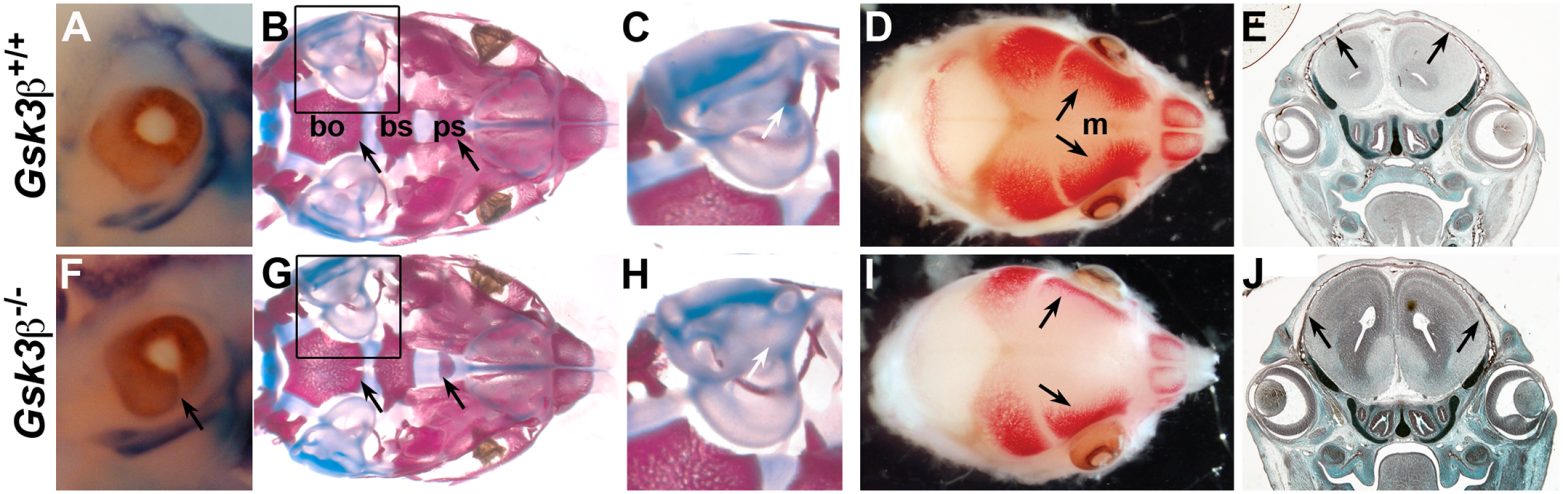
Deletion of *Gsk3β* results in ocular, cranial base and skull vault defects. (A-E) Control *Gsk3β* ^+/+^ mice. F-J) *GS*K3*β* ^*-/-*^ mice. Alizarin red staining marks the bone and alcian blue staining marks the cartilage. (A, F) Loss of *Gsk3β* results in ocular coloboma (F, arrow). (B, G) At E18.5, in the cranial base, the basioccipital and basisphenoid are cleft and the presphenoid is smaller (arrows, G). (C, H) Ossification of the ear is delayed in the mutant (arrow, H). (D, I) At E15.5 the frontal bone is smaller with a concomitant increase in the width of the metopic suture (m). (E, J) Coronal sections at E15.5. Mutant frontal bones (in J) are smaller than in wildtype (in E). Arrows mark apical extent of frontal bones. bo, basioccipital; bs, basisphenoid; f, frontal; m, metopic; p, parietal; ps, presphenoid.

In the mouse, deletion of both *Gsk3* genes is catastrophic, resulting in pre-implantation lethality [36]. This is unsurprising, given the reported ubiquitous expression of both genes [37]. Since then, we have shown that the phenotypes in single knockouts of *Gsk3α* and *Gsk3β* are very different, suggesting tissue specific functions of the two genes [11,18]. *Gsk3αa* is dispensible for life [11,17]. While original knockout of *Gsk3β* (*Gsk3β*^*tm1Jrw*^) appeared to be lethal at mid-gestation stages [20], multiple recent reports have shown that *Gsk3β* mutants die at birth, from a complete cleft of the secondary palate [11,18,19,21,36]. However, other associated cranial phenotypes have not been well documented. Thus, we examined the phenotypes of all three *Gsk3β* null alleles during embryogenesis. As previously noted, all three alleles had a fully penetrant cleft secondary palate [11,18] and data not shown). Externally, the most obvious phenotype was ocular coloboma (Fig 1F). The cranial base was also cleft, with diminished ossification of the presphenoid (PS) (compare Fig 1G to 1B) and a reduction in ossification of the inner ear bones (compare Fig 1H to 1C). Finally, we observed malformations of the skull vault; specifically, the frontal bones are smaller compared to control littermates at E15.5, leading to an enlarged fontanelle (compare Fig 1I, 1J to 1D, 1E).

### Gs*k3* mRNAs and GSK3 proteins are expressed in the frontal bone primordia

**Fig 2:**
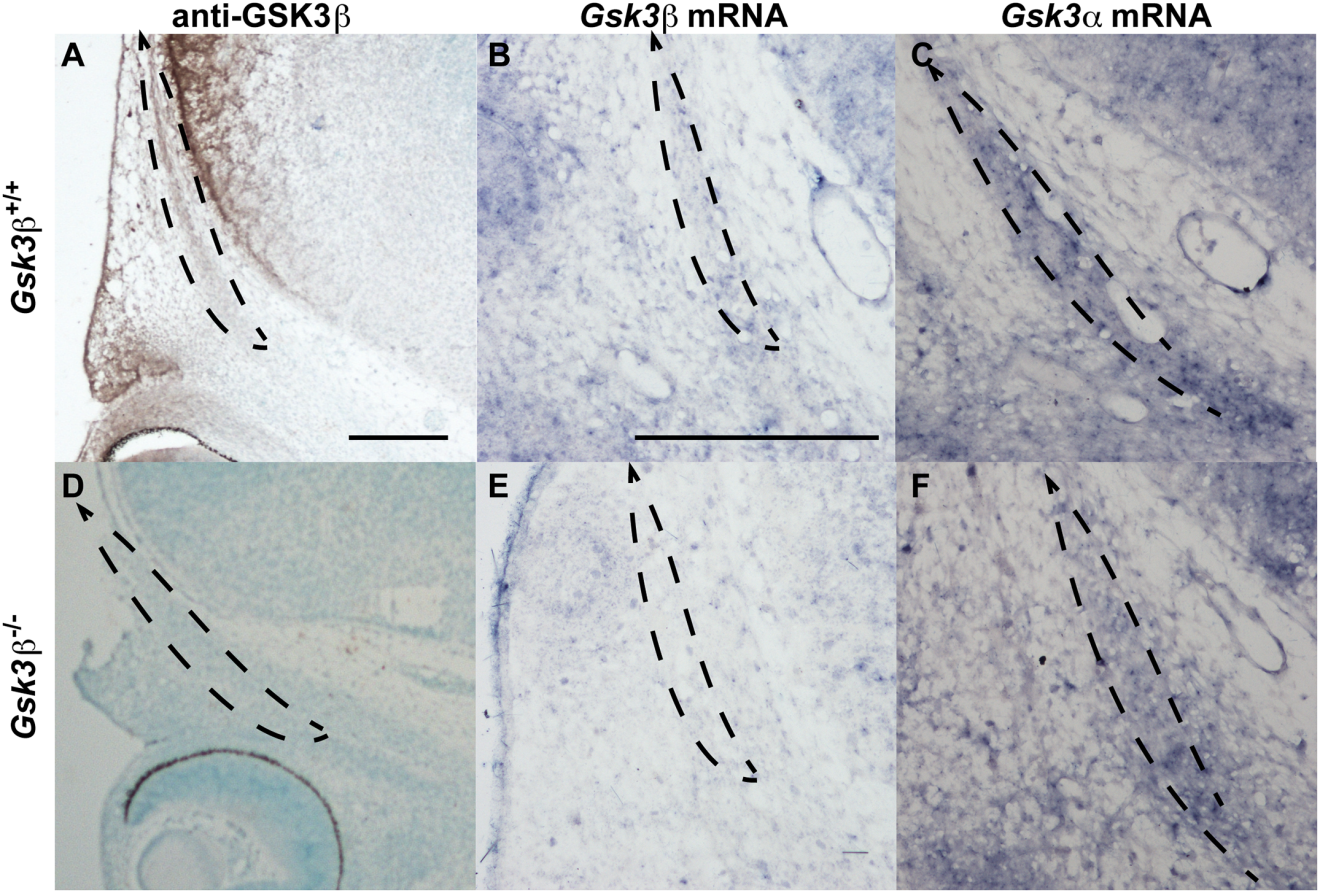
*Gsk3β* is expressed in the E13.5 frontal bones, and *Gsk3α* expression is not affected in *Gsk3β* mutants. Coronal cross-sections of E13.5 mouse heads, through the frontal bones (outlined by dotted lines). **(A-C)** E13.5 control embryos stained for GSK3β protein (A), and mRNA expression (B). *Gsk3α* mRNA is also found in the frontal bone primordia (C). **(D-F)** In *Gsk3β* mutants, we do not observe any residual GSK3β protein (D), or mRNA (E). There is no obvious change in *Gsk3α* mRNA expression in the *Gsk3β* mutant frontal bone (F). Scale bars = 100 mm, A applies to D; B applies to C, E, F.

Because of the clear decrease in frontal bone size in *Gsk3β* mutants, we decided to examine expression of both *Gsk3α* and *Gsk3β* in the condensing mesenchyme destined to form the frontal bone. Both GSK3B protein and transcript are expressed in frontal bone primordia at E13.5 (Fig 2A and 2B). Importantly, Gs*k3β* mRNA expression is absent in the *Gsk3β* mutant (Fig 2E). Although *Gsk3α* is also expressed in the E13.5 frontal bone, we found that this expression was unchanged in the *Gsk3β* mutant animals (Fig 2F).

### Neural crest migration follows appropriate paths but is reduced in the *Gsk3β*^*-/-*^ mutants

**Fig 3:**
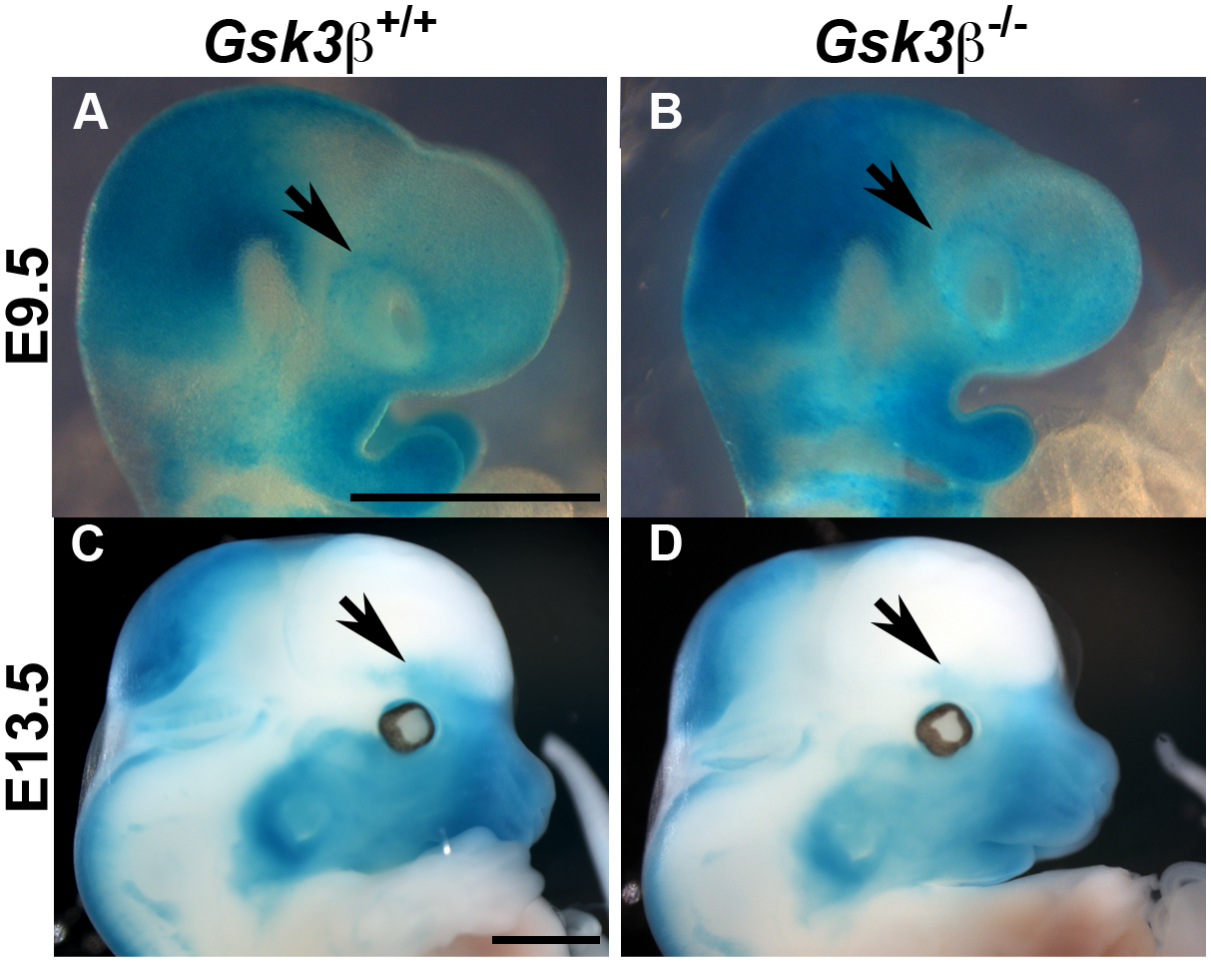
The neural crest derived frontal bone primordia in *Gsk3β* mutants is reduced by E13.5. Lateral views of *LacZ* staining (in blue) marks the Wnt1::cre positive neural crest population. Cranial regions are shown in E9.5 mouse embryos (A, B) and at E13.5 (C, D). (A, B) At E9.5, cranial neural crest in controls (A) and mutants (B) appear similar (n = 4/4 mutants). (C, D) At E13.5, the mutant neural crest derived frontal bone condensation (D, arrow) is smaller than wildtype littermate (n = 2/2 mutants C, arrow). Scale bars = 1 mm.

As mentioned above, the frontal bones are formed from neural crest derived mesenchyme [1,38]. Therefore, we considered the possibility that defects in neural crest migration or cell number could lead to a smaller condensation. To test this, we performed a lineage tracing experiment using the neural crest specific driver Wnt1::cre combined with the R26R^lacZ^ reporter [25,26]. We found only subtle changes in the neural crest cells. At e9.5 migration appeared normal, with similar levels of positive cells adjacent to the eye (n = 4/4 mutants; black arrows, Fig 3A and 3B). However, by E13.5, there appears to be a decrease in the β-galactosidase activity in frontal bone condensation in mutant animals (n = 2/2 mutants; blue staining, black arrows, Fig 3D). This suggests that the frontal bone phenotype arises in the condensing neural crest cells after E9.5 and before E13.5.

### At E12.5, mutant frontal condensations express appropriate osteoblast markers

**Fig 4:**
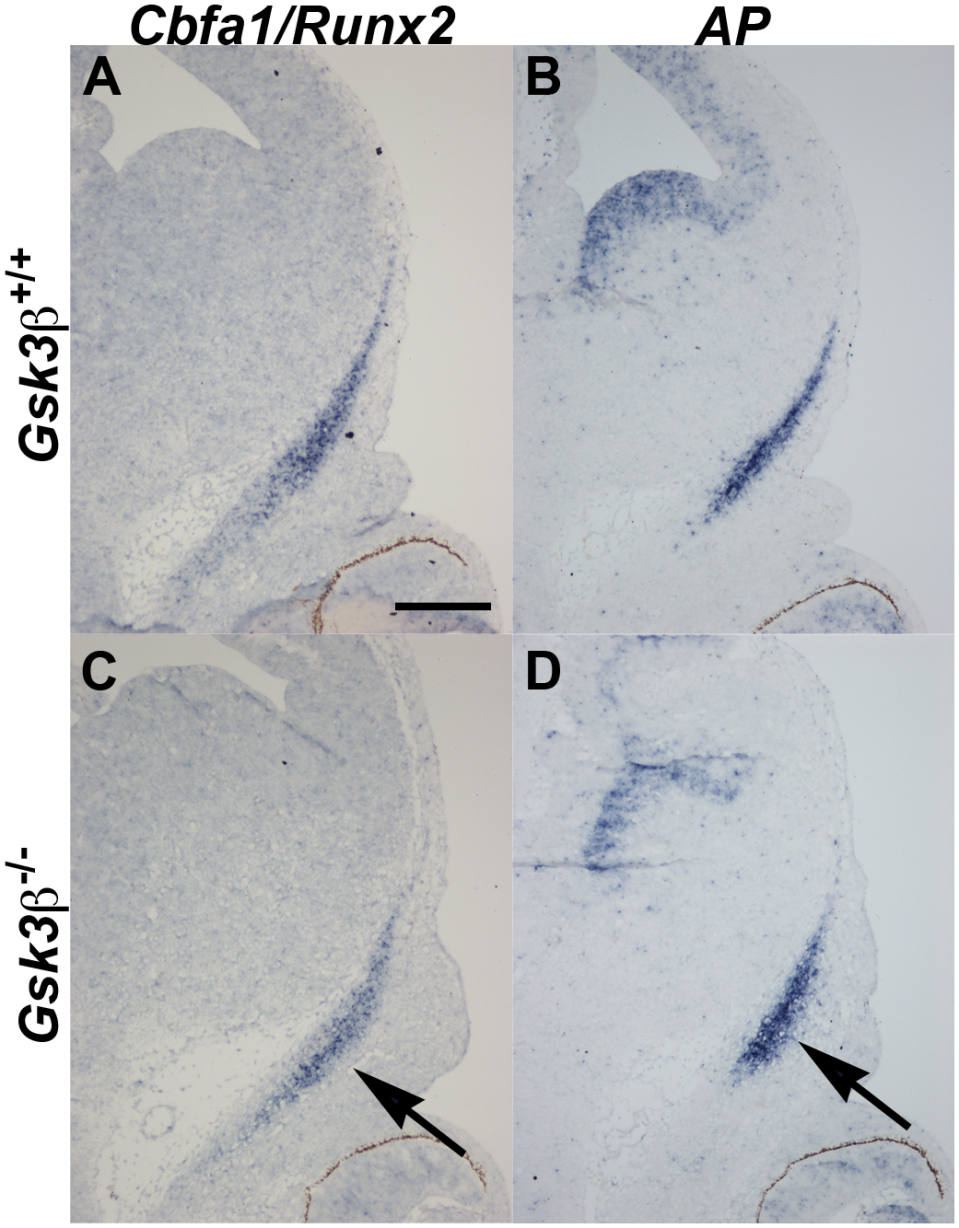
Osteogenic differentiation is occurring in the smaller frontal bone primordium at E12.5. mRNA *in situ* hybridization on coronal cross-sections through the frontal bone primordia (outlined in yellow). (A, B) *Gsk*3*β*^*+/+*^ animals. (C, D) *Gsk*3*β*^*-/-*^ mutants. (A, C) *Cbfa1/Runx2* mRNA *in situ* hybridization. (B, D) *Alkalkine phosphatase (AP)* mRNA *in situ* hybridization. (C, D) Note that the domain of expression of both genes is marginally smaller in the *Gsk3β* null animals. Scale bar = 100 mm.

We then considered whether differentiation of osteoblasts was occurring at the right time and in the right place. To do this, we performed mRNA *in situ* hybridization at E12.5, when the condensations are histologically similar in mutants and controls. First, we examined expression of *Cbfa1/Runx2* and *alkaline phosphatase (AP)*. We found that although there was no significant difference in intensity in the *in situ* signals, both *Cbfa1* and *AP* domains are misshapen (n = 3/genotype; Fig 4C and 4D, arrow). The mutant *AP* and *Cbfa1* domains do not extend as far apically and are expanded in the mediolateral domain compared to the wildtype littermate control (Fig 4C and 4D). Both control and mutant frontal bones also express *Msx1* and *Msx2* in appropriate, but smaller domains (data not shown). We concluded that although slightly diminished, mutant bones are undergoing appropriate osteoblastic differentiation at E12.5.

### Loss of *Gsk3β* triggers premature differentiation at E13.5

**Fig 5:**
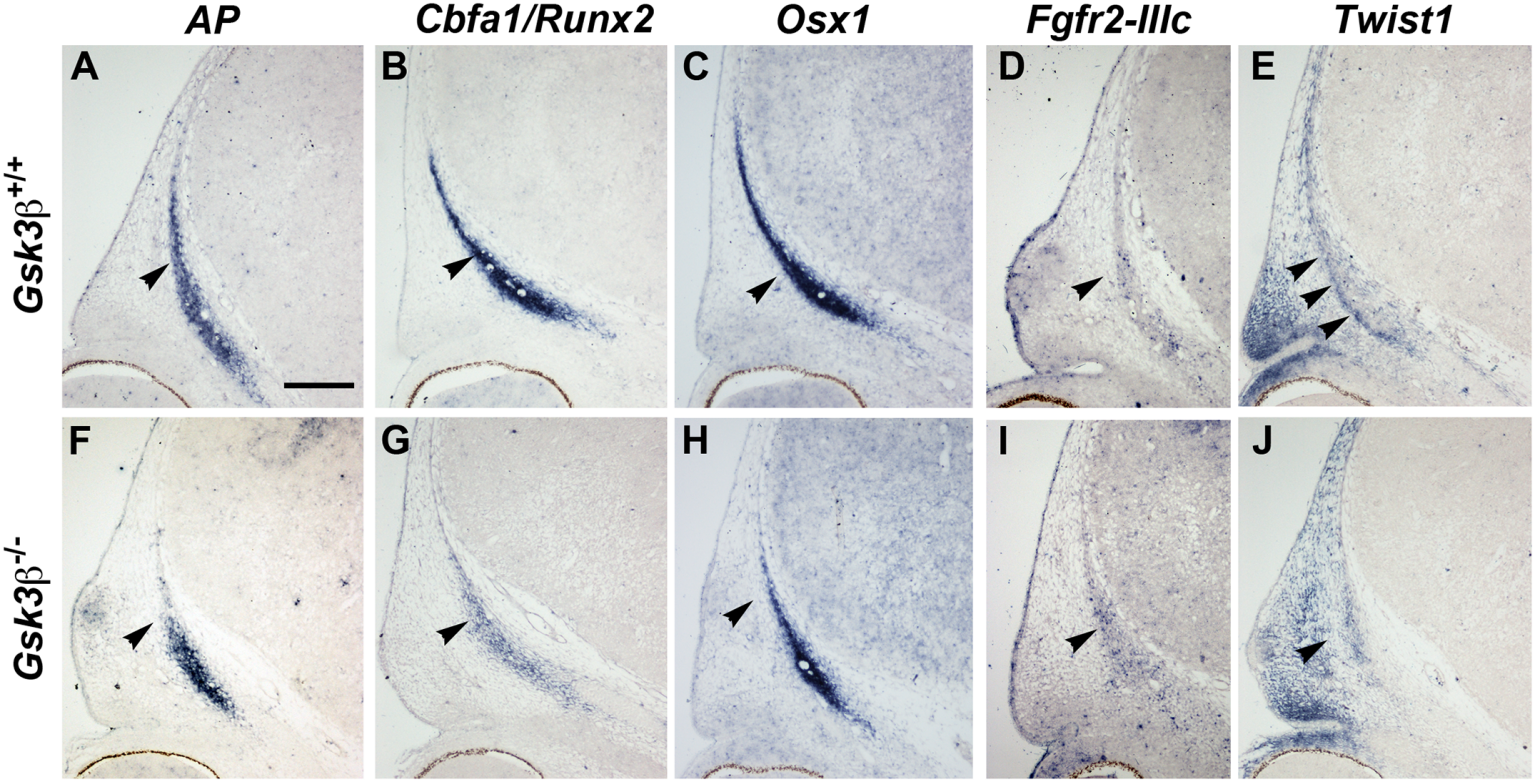
Disorganized frontal bone differentiation in *Gsk3β* mutants. mRNA *in situ* hybridization for indicated mRNAs on coronal cross-sections through the condensing frontal bone. (A-E) *Gsk3β*^*+/+*^ animals. (F-J) *Gsk3β*^*-/-*^ mutants. (A-B, F-G) The frontal bone condensation expresses *AP* (F) and *Cbfa1/Runx2* (G) in both wildtype and mutant littermates. Mutant condensations (B, G) remain smaller. (C, H) There is no increase in the Wnt dependent osteogenic gene *Osx1*, which is expressed normally in mutant frontal bone (H). (D, I) *Fgfr2-IIIc* is upregulated in the mutant frontal bone (I, arrow). (E, J) In wildtype animals (E) *Twist1* expression marks the ectocranial edge of the frontal bone condensation (arrowheads, E), and borders on an adjacent *Twist1*-negative region. (J) In mutants, *Twist1* is expanded diffusely, leading to an absence of a clearly demarcated, *Twist*-positive ectocranial border. Scale bar = 100 mm.

By E13.5, the frontal bone compartments were markedly different between mutant animals and littermate controls (Fig 5). These data suggested that GSK3β is critically important between E12.5 and E13.5. At these stages, both wildtype and mutant condensations continue to express *AP* in the appropriate domains (Fig 5A and 5F), with some decrease in *Cbfa1* (Fig 5F and 5G). We hypothesized that in the mutant animals, frontal bone osteoblasts might be differentiating prematurely, rather than maintaining a growth and expansion phase. To test this idea, we looked at markers of osteogenic differentiation, *Fgfr2-IIIc* and *Twist1*, by mRNA *in situ* hybridization. We observed that *Fgfr2-IIIc* expression was significantly upregulated in the mutant frontal bone (Fig 5I). Correlating with the increased *Fgfr2-IIIc* expression, we also noted a change in the domain of *Twist1* expression (Fig 5E and 5J). In the wildtype situation, a stripe of *Twist1* expression at the ectocranial border of the frontal condensation distinguishes the frontal bone anlagen from the dermis (arrowheads, Fig 5E). In mutant embryos, we observed that the mesenchyme is not divided into these two compartments; instead, *Twist1* expression expands throughout (Fig 5J). From these data we conclude that the subsequent reduced ossification in the mutants is associated with either premature differentiation or aberrant compartmentalization of the frontal bone anlagen.

### Changes in *Gsk3β* mutants are not due to Wnt signaling

**Fig 6:**
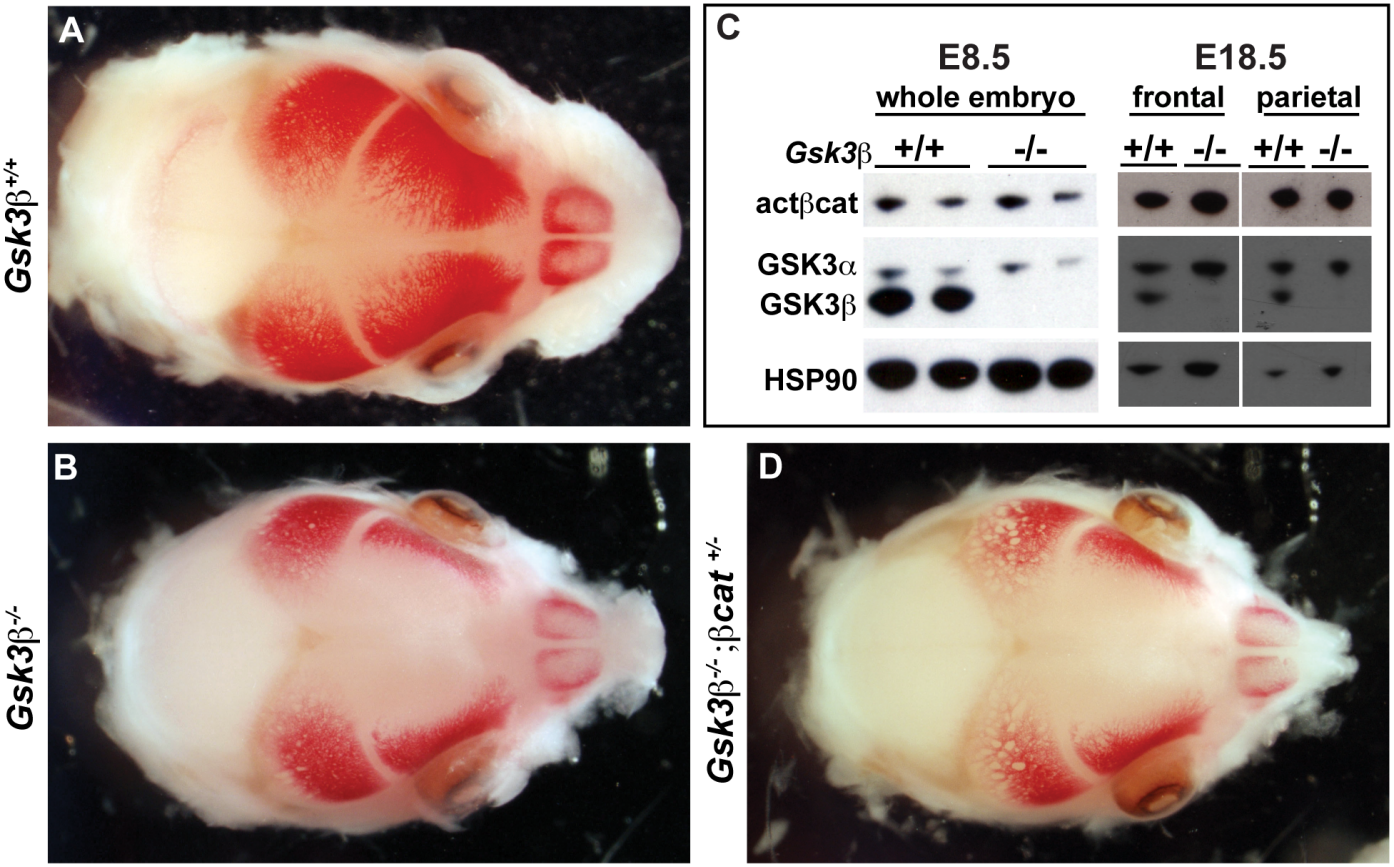
Skull phenotypes in *Gsk3β* mutants are not due to increase activated β-catenin. (A, B and D) Alizarin red staining marks the calvarial bones at E15.5. (A) *Gsk*3*β*^*+/+*^ animals. (B) *Gsk*3*β*^*-/-*^ mutants. (D) *Gsk*3*β*^*-/-*^; *β-catenin*^*+/-*^ mutants. (C) Western blot analysis of protein lysates from whole E8.5 embryos (left lanes) and E18.5 frontal and parietal bones (right lanes). We observed no difference in the amount activated *β*-catenin (act*b*cat), or GSK3α in the mutant animals. HSP-90 (heat shock protein-90) is a loading control. Note that null alleles of *Gsk3β* produce no detectable protein.

Since loss of GSK3β function is predicted to increase the amount of activated β-catenin in the embryo, we tested whether expression of the Wnt targets *Osterix-1* (*Osx1)* and *Axin2* was increased in the mutants. Both markers showed no change in the levels of expression in the frontal bone primordia (compare Fig 5C to 5H, and data not shown). We also tested whether the levels of activated β-catenin was changed in the mutants. We found that *Gsk3β* mutants had no difference in the amount of activated β-catenin at E8.5 embryos or in E18.5 frontal and parietal bones (Fig 6C). Furthermore, heterozygosity of *β-catenin* did not rescue the wide fontanelle at E15.5 (compare Fig 6D to 6B). However, the size of the *Osx1* domain is much smaller, consistent with the diminished size and shape of the overall condensation.

### Decreased proliferation and increased cell death in the frontal bone condensation

**Fig 7:**
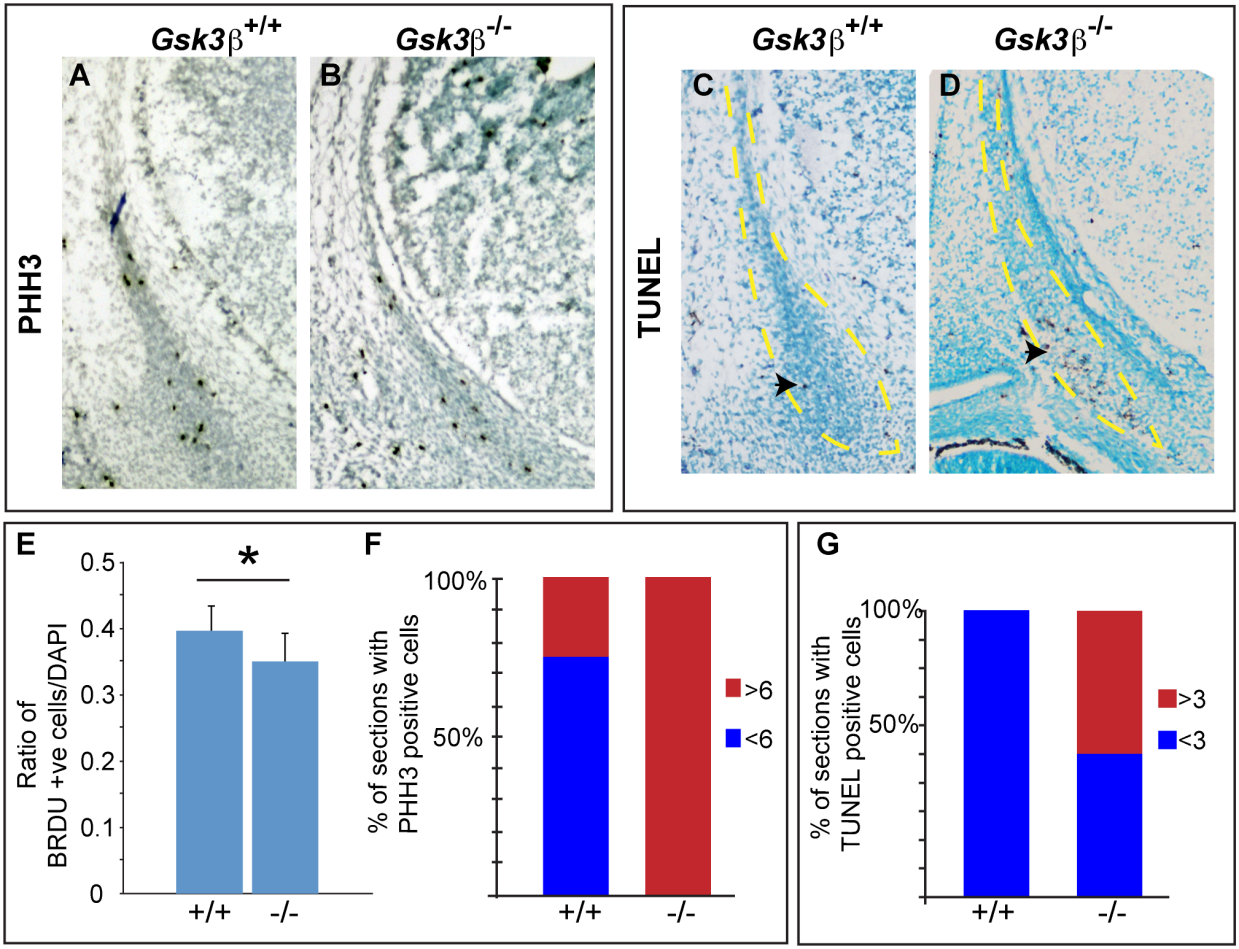
Loss of *Gsk3β* leads to decreased proliferation and increased cell death in the frontal bone primordia. (A-D) Coronal cross-sections through the condensing frontal bone, outlined in yellow. (A-B) Mitotic cells were detected by antibody staining detecting phosphorylated histone H3 (pHH3). Mutant sections showed more mitotic cells (B). (C-D) Cell death was detected by TUNEL staining. Mutant sections showed increased cell death (D), (E) BRDU staining revealed a small but significantly lower ratio of cells in S-phase in the mutant frontal bone (p <0.05). (F) Antibody staining for pHH3 positive cells showed more mitotic cells in the mutants. Slides were scored with sections with greater than six (red) or less than six positive cells (blue). (G) All *Gsk3β*^*+/+*^ frontal bone sections had fewer than three apoptotic cells, while the *Gsk3β*^*-/-*^ animals showed increase in cell death, based on the number of cells that have TUNEL-positive staining.

Finally, we thought that premature differentiation might be accompanied by decreased proliferation. To test this, we examined the number of cells in S-phase by pulsing animals with bromo-deoxyuridine (BrdU). We also counted mitotic cells by labeling with a phosphorylated histone H3 (PHH3) antibody. At E13.5 we found a decreased number of cells in S-phase via BrdU staining in the frontal bone (Fig 7E, p<0.05). Surprisingly, we observed an increased number of mitotic cells (Fig 7B and 7F). As GSK3 is known to phosphorylate p27kip1 [39,40], one possibility is that the loss of GSK3β leads to a mild arrest at the G1/S checkpoint. We also considered the possibility that there could be increased cell death in the mutants. Indeed, TUNEL assays revealed more cell death in the mutant frontal bones (Fig 7C). Thus, we observe precocious differentiation and decreased cell numbers in *Gsk3β* mutants. Taken together, these two mechanisms lead to an overall reduction in the pool of osteoprogenitor cells and a smaller frontal bone.

## Discussion

Pathological changes in skull development are among the most frequent congenital anomalies associated with live births; thus, calvarial perturbations present a major medical challenge[23]. Insufficient cranial bone is frequently attributed to brain abnormalities [41,42]; however, in recent years, it has become clear that mutation in a number of key genes can disrupt the progression of the intrinsic ossification programmes. Here, we demonstrate a requirement for *Gsk3β* in the initiation and expansion of the frontal bone primordium. We find that *Gsk3β* mutants display premature osteoblastic differentiation in the frontal bone compartment. This, combined with changes in cell proliferation and increased cell death, leads to smaller frontal bones and a wide fontanelle.

We also considered the possibility that the smaller osteoblastic compartment could arise due to neural crest migration defects. Both *Xenopus* Twist1 and mammalian Snail1 proteins are reported to be Gs*k3* substrates; these are key regulators of neural crest cell migration [16,43–45]. Embryos with a complete loss of *Twist1 (Twist1*^*-/-*^) have a severe cranial phenotype, stemming from earlier defects in cranial neural crest migration [46,47]. This is consistent with an evolutionarily conserved role for *Twist1* during the critical migratory stages, which then obscures a later role in the ossification and migration of the neural crest derived skull vault mesenchyme. *Snail1* mutation also leads to midgestational lethality and thus, roles in the skull are unclear [48,49]. Using lineage tracing to examine migration of the neural crest, we noted no significant changes in the frontal bone-directed cranial neural crest. This may reflect functional redundancy between mammalian Gs*k3α* and Gs*k3β* which warrants further study.

*Twist1* heterozygotes (*Twist1*^*+/-*^) develop coronal craniosynostosis, owing in part to abberant migration of Wnt-1cre positive cells into the mesodermal compartment of the coronal suture [50–52]. Coronal synostosis in the *Twist1* heterozygotes is thought to result from a switch of Twist/E2A heterodimers in wildtype animals to Twist homodimers. Twist homodimers preferentially upregulate expression of *FGFR2* and subsequent differentiation at the osteogenic front [53,54]. In our studies, the loss of compartmentalization of the *Twist1* expression domain may also prevent preosteoblasts from migrating and populating the growing osteogenic front. Instead, pre-osteoblasts may differentiate *in situ* in the forming frontal bone anlagen. Though we cannot exclude subtle defects in cell migration, precocious differentiation of osteoblast precursors in the frontal bone primordia will certainly lead to a smaller frontal bone.

The defects we observe in the *Gsk3β* mutant skulls are more similar to two other mouse models: transgenic dominant negative BMPR1a, and compound *Msx2*^*+/-*^*; Twist1*^*+/-*^ mutants [22,55]. Several reports suggest that human BMP receptor 1A mutations also lead to craniofacial dysmorphism [56,57], and mutations in human MSX2 lead to persistent calvarial foramina [58]. In the mouse, expression of dominant-negative Bmpr1a in the neural crest leads to severe apoptosis of the frontal bone primordia accompanied by facial clefting [55]. Similarly, enlarged foramina are present in human Saethre-Chotzen patients [59]. In the mouse models for Saethre-Chotzen syndrome (single or compound *Msx2-Twist* mutants), osteoblastic differentiation markers were reduced by E12.5, while proliferation is not reduced until E14.5 [22]. In contrast, our data suggest a novel etiology for smaller skull vault bone formation, namely premature expression of differentiation markers, and changes in the cell cycle. This implies an acceleration of the ossification programme, leading to a depletion of the osteoblastic progenitor compartment of the frontal bone.

Finally, we considered the possibility that *Gsk3β* is required for a Wnt/β-catenin dependent function during development of the neural crest derived skull. As we saw no change in Wnt-dependent target genes such as *Axin2*, and we found no rescue when decreasing the genetic dose of *β-catenin* (Fig 6), we propose that these functions of *Gsk3β* are *β-catenin*-independent. *Gsk3α* expression may be sufficient to compensate for *Gsk3β* in Wnt signaling, especially given the critically important roles for Wnt signaling in stem cell maintenance and early development [36]. However, it is worth noting that postnatal deletion of *Gsk3β* in osteoblasts appears sufficient to increase levels of activated β-catenin [60]. Furthermore, *Gsk3β* has been reported to phosphorylate and inactivate Cbfa1/Runx2 [21]. Both of these observations could reflect a difference in the prenatal intramembranous ossification programme versus postnatal Wnt-dependent ossification programmes. As the majority of craniofacial congenital anomalies manifest *in utero*, future studies should focus on distinguishing between temporal and tissue specific substrates of *Gsk3*.

## References

[1] Yoshida T, Vivatbutsiri P, Morriss-Kay G, Saga Y, Iseki S (2008) Cell lineage in mammalian craniofacial mesenchyme. Mech Dev 125: 797–808. 10.1016/j.mod.2008.06.007 18617001

[2] Yoshida T (2005) [Growth pattern of the frontal bone primordium and involvement of Bmps in this process]. Kokubyo Gakkai Zasshi 72: 19–27. 15856768 10.5357/koubyou.71and72.19

[3] Richtsmeier JT, Flaherty K (2013) Hand in glove: brain and skull in development and dysmorphogenesis. Acta Neuropathol 125: 469–489. 10.1007/s00401-013-1104-y 23525521 PMC3652528

[4] Day TF, Guo X, Garrett-Beal L, Yang Y (2005) Wnt/beta-catenin signaling in mesenchymal progenitors controls osteoblast and chondrocyte differentiation during vertebrate skeletogenesis. Dev Cell 8: 739–750. 15866164 10.1016/j.devcel.2005.03.016

[5] Goodnough LH, Dinuoscio GJ, Ferguson JW, Williams T, Lang RA, Atit RP (2014) Distinct requirements for cranial ectoderm and mesenchyme-derived wnts in specification and differentiation of osteoblast and dermal progenitors. PLoS Genet 10: e1004152 10.1371/journal.pgen.1004152 24586192 PMC3930509

[6] Glass DA 2nd, Bialek P, Ahn JD, Starbuck M, Patel MS, Clevers H, et al (2005) Canonical Wnt signaling in differentiated osteoblasts controls osteoclast differentiation. Dev Cell 8: 751–764. 15866165 10.1016/j.devcel.2005.02.017

[7] Hill TP, Spater D, Taketo MM, Birchmeier W, Hartmann C (2005) Canonical Wnt/beta-catenin signaling prevents osteoblasts from differentiating into chondrocytes. Dev Cell 8: 727–738. 15866163 10.1016/j.devcel.2005.02.013

[8] Behr B, Longaker MT, Quarto N (2013) Absence of endochondral ossification and craniosynostosis in posterior frontal cranial sutures of Axin2(-/-) mice. PLoS One 8: e70240 10.1371/journal.pone.0070240 23936395 PMC3731366

[9] Liu B, Yu HM, Hsu W (2007) Craniosynostosis caused by Axin2 deficiency is mediated through distinct functions of beta-catenin in proliferation and differentiation. Dev Biol 301: 298–308. 17113065 10.1016/j.physletb.2003.10.071 PMC1821096

[10] Yu HM, Jerchow B, Sheu TJ, Liu B, Costantini F, Puzas JE, et al (2005) The role of Axin2 in calvarial morphogenesis and craniosynostosis. Development 132: 1995–2005. 15790973 10.1242/dev.01786 PMC1828115

[11] Barrell WB, Szabo-Rogers HL, Liu KJ (2012) Novel reporter alleles of GSK-3alpha and GSK-3beta. PLoS One 7: e50422 10.1371/journal.pone.0050422 23185619 PMC3503927

[12] Jia J, Amanai K, Wang G, Tang J, Wang B, Jiang J (2002) Shaggy/GSK3 antagonizes Hedgehog signalling by regulating Cubitus interruptus. Nature 416: 548–552. 11912487 10.1038/nature733

[13] Price MA, Kalderon D (2002) Proteolysis of the Hedgehog signaling effector Cubitus interruptus requires phosphorylation by Glycogen Synthase Kinase 3 and Casein Kinase 1. Cell 108: 823–835. 11955435 10.1016/s0092-8674(02)00664-5

[14] Kim WY, Wang X, Wu Y, Doble BW, Patel S, Woodgett JR, et al (2009) GSK-3 is a master regulator of neural progenitor homeostasis. Nat Neurosci 12: 1390–1397. 10.1038/nn.2408 19801986 PMC5328673

[15] Liberman Z, Eldar-Finkelman H (2005) Serine 332 phosphorylation of insulin receptor substrate-1 by glycogen synthase kinase-3 attenuates insulin signaling. J Biol Chem 280: 4422–4428. 15574412 10.1074/jbc.M410610200

[16] Lander R, Nasr T, Ochoa SD, Nordin K, Prasad MS, Labonne C (2013) Interactions between Twist and other core epithelial-mesenchymal transition factors are controlled by GSK3-mediated phosphorylation. Nat Commun 4: 1542 10.1038/ncomms2543 23443570 PMC4198179

[17] MacAulay K, Doble BW, Patel S, Hansotia T, Sinclair EM, Drucker DJ, et al (2007) Glycogen synthase kinase 3alpha-specific regulation of murine hepatic glycogen metabolism. Cell Metab 6: 329–337. 17908561 10.1016/j.cmet.2007.08.013

[18] Liu KJ, Arron JR, Stankunas K, Crabtree GR, Longaker MT (2007) Chemical rescue of cleft palate and midline defects in conditional GSK-3beta mice. Nature 446: 79–82. 17293880 10.1038/nature05557

[19] Kerkela R, Kockeritz L, Macaulay K, Zhou J, Doble BW, Beahm C, et al (2008) Deletion of GSK-3beta in mice leads to hypertrophic cardiomyopathy secondary to cardiomyoblast hyperproliferation. J Clin Invest 118: 3609–3618. 10.1172/JCI36245 18830417 PMC2556242

[20] Hoeflich KP, Luo J, Rubie EA, Tsao MS, Jin O, Woodgett JR (2000) Requirement for glycogen synthase kinase-3beta in cell survival and NF-kappaB activation. Nature 406: 86–90. 10894547 10.1038/35017574

[21] Kugimiya F, Kawaguchi H, Ohba S, Kawamura N, Hirata M, Chikuda H, et al (2007) GSK-3beta controls osteogenesis through regulating Runx2 activity. PLoS One 2: e837 17786208 10.1371/journal.pone.0000837 PMC1950686

[22] Ishii M, Merrill AE, Chan YS, Gitelman I, Rice DP, Sucov HM, et al (2003) Msx2 and Twist cooperatively control the development of the neural crest-derived skeletogenic mesenchyme of the murine skull vault. Development 130: 6131–6142. 14597577 10.1242/dev.00793

[23] Johnson D, Wilkie AO (2011) Craniosynostosis. Eur J Hum Genet 19: 369–376. 10.1038/ejhg.2010.235 21248745 PMC3060331

[24] Stankunas K, Bayle JH, Gestwicki JE, Lin YM, Wandless TJ, Crabtree GR (2003) Conditional protein alleles using knockin mice and a chemical inducer of dimerization. Mol Cell 12: 1615–1624. 14690613 10.1016/s1097-2765(03)00491-x

[25] Danielian PS, Muccino D, Rowitch DH, Michael SK, McMahon AP (1998) Modification of gene activity in mouse embryos in utero by a tamoxifen-inducible form of Cre recombinase. Curr Biol 8: 1323–1326. 9843687 10.1016/s0960-9822(07)00562-3

[26] Soriano P (1999) Generalized lacZ expression with the ROSA26 Cre reporter strain. Nat Genet 21: 70–71. 9916792 10.1038/5007

[27] Tabler JM, Barrell WB, Szabo-Rogers HL, Healy C, Yeung Y, Perdiguero EG, et al (2013) Fuz mutant mice reveal shared mechanisms between ciliopathies and FGF-related syndromes. Dev Cell 25: 623–635. 10.1016/j.devcel.2013.05.021 23806618 PMC3697100

[28] Lewandoski M, Martin GR (1997) Cre-mediated chromosome loss in mice. Nat Genet 17: 223–225. 9326948 10.1038/ng1097-223

[29] Huelsken J, Vogel R, Erdmann B, Cotsarelis G, Birchmeier W (2001) beta-Catenin controls hair follicle morphogenesis and stem cell differentiation in the skin. Cell 105: 533–545. 11371349 10.1016/s0092-8674(01)00336-1

[30] Winslow MM, Pan M, Starbuck M, Gallo EM, Deng L, Karsenty G, et al (2006) Calcineurin/NFAT signaling in osteoblasts regulates bone mass. Dev Cell 10: 771–782. 16740479 10.1016/j.devcel.2006.04.006

[31] Yaguchi Y, Yu T, Ahmed MU, Berry M, Mason I, Basson MA (2009) Fibroblast growth factor (FGF) gene expression in the developing cerebellum suggests multiple roles for FGF signaling during cerebellar morphogenesis and development. Dev Dyn 238: 2058–2072. 10.1002/dvdy.22013 19544582

[32] Fuchtbauer EM (1995) Expression of M-twist during postimplantation development of the mouse. Dev Dyn 204: 316–322. 8573722 10.1002/aja.1002040309

[33] Thomas BL, Sharpe PT (1998) Patterning of the murine dentition by homeobox genes. Eur J Oral Sci 106 Suppl 1: 48–54. 9541203 10.1111/j.1600-0722.1998.tb02153.x

[34] Ashique AM, Fu K, Richman JM (2002) Signalling via type IA and type IB bone morphogenetic protein receptors (BMPR) regulates intramembranous bone formation, chondrogenesis and feather formation in the chicken embryo. Int J Dev Biol 46: 243–253. 11934153

[35] Szabo-Rogers HL, Geetha-Loganathan P, Nimmagadda S, Fu KK, Richman JM (2008) FGF signals from the nasal pit are necessary for normal facial morphogenesis. Dev Biol 318: 289–302. 10.1016/j.ydbio.2008.03.027 18455717

[36] Doble BW, Patel S, Wood GA, Kockeritz LK, Woodgett JR (2007) Functional redundancy of GSK-3alpha and GSK-3beta in Wnt/beta-catenin signaling shown by using an allelic series of embryonic stem cell lines. Dev Cell 12: 957–971. 17543867 10.1016/j.devcel.2007.04.001 PMC4485918

[37] Woodgett JR (1990) Molecular cloning and expression of glycogen synthase kinase-3/factor A. EMBO J 9: 2431–2438. 2164470 10.1002/j.1460-2075.1990.tb07419.x PMC552268

[38] Jiang X, Iseki S, Maxson RE, Sucov HM, Morriss-Kay GM (2002) Tissue origins and interactions in the mammalian skull vault. Dev Biol 241: 106–116. 11784098 10.1006/dbio.2001.0487

[39] Surjit M, Lal SK (2007) Glycogen synthase kinase-3 phosphorylates and regulates the stability of p27kip1 protein. Cell Cycle 6: 580–588. 17351340 10.4161/cc.6.5.3899

[40] Wang Q, Zhou Y, Wang X, Evers BM (2008) p27 Kip1 nuclear localization and cyclin-dependent kinase inhibitory activity are regulated by glycogen synthase kinase-3 in human colon cancer cells. Cell Death Differ 15: 908–919. 10.1038/cdd.2008.2 18408738 PMC2432084

[41] Sun JK, LeMay DR, Couldwell WT, Zee C (1995) Frontal foramina in pediatric skull in cases of congenital hydrocephalus. Radiology 197: 497–499. 7480701 10.1148/radiology.197.2.7480701

[42] Reimao R, Plaggert PG, Adda C, Matushita H, Reed UC (2003) Frontal foramina, Chiari II malformation, and hydrocephalus in a female. Pediatr Neurol 29: 341–344. 14643399 10.1016/s0887-8994(03)00279-0

[43] Yook JI, Li XY, Ota I, Fearon ER, Weiss SJ (2005) Wnt-dependent regulation of the E-cadherin repressor snail. J Biol Chem 280: 11740–11748. 15647282 10.1074/jbc.M413878200

[44] Yook JI, Li XY, Ota I, Hu C, Kim HS, Kim NH, et al (2006) A Wnt-Axin2-GSK3beta cascade regulates Snail1 activity in breast cancer cells. Nat Cell Biol 8: 1398–1406. 17072303 10.1038/ncb1508

[45] Zhou BP, Deng J, Xia W, Xu J, Li YM, Gunduz M, et al (2004) Dual regulation of Snail by GSK-3beta-mediated phosphorylation in control of epithelial-mesenchymal transition. Nat Cell Biol 6: 931–940. 15448698 10.1038/ncb1173

[46] Chen ZF, Behringer RR (1995) twist is required in head mesenchyme for cranial neural tube morphogenesis. Genes Dev 9: 686–699. 7729687 10.1101/gad.9.6.686

[47] Soo K, O'Rourke MP, Khoo PL, Steiner KA, Wong N, Behringer RR, et al (2002) Twist function is required for the morphogenesis of the cephalic neural tube and the differentiation of the cranial neural crest cells in the mouse embryo. Dev Biol 247: 251–270. 12086465 10.1006/dbio.2002.0699

[48] Murray SA, Gridley T (2006) Snail1 gene function during early embryo patterning in mice. Cell Cycle 5: 2566–2570. 17106264 10.4161/cc.5.22.3502

[49] Murray SA, Gridley T (2006) Snail family genes are required for left-right asymmetry determination, but not neural crest formation, in mice. Proc Natl Acad Sci U S A 103: 10300–10304. 16801545 10.1073/pnas.0602234103 PMC1502452

[50] Bialek P, Kern B, Yang X, Schrock M, Sosic D, Hong N, et al (2004) A twist code determines the onset of osteoblast differentiation. Dev Cell 6: 423–435. 15030764 10.1016/s1534-5807(04)00058-9

[51] Bildsoe H, Loebel DA, Jones VJ, Chen YT, Behringer RR, Tam PP (2009) Requirement for Twist1 in frontonasal and skull vault development in the mouse embryo. Dev Biol 331: 176–188. 10.1016/j.ydbio.2009.04.034 19414008

[52] Ting MC, Wu NL, Roybal PG, Sun J, Liu L, Yen Y, et al (2009) EphA4 as an effector of Twist1 in the guidance of osteogenic precursor cells during calvarial bone growth and in craniosynostosis. Development 136: 855–864. 10.1242/dev.028605 19201948 PMC2685950

[53] Connerney J, Andreeva V, Leshem Y, Mercado MA, Dowell K, Yang X, et al (2008) Twist1 homodimers enhance FGF responsiveness of the cranial sutures and promote suture closure. Dev Biol 318: 323–334. 10.1016/j.ydbio.2008.03.037 18471809 PMC2605972

[54] Connerney J, Andreeva V, Leshem Y, Muentener C, Mercado MA, Spicer DB (2006) Twist1 dimer selection regulates cranial suture patterning and fusion. Dev Dyn 235: 1345–1357. 16502419 10.1002/dvdy.20717

[55] Saito H, Yamamura K, Suzuki N (2012) Reduced bone morphogenetic protein receptor type 1A signaling in neural-crest-derived cells causes facial dysmorphism. Dis Model Mech 5: 948–955. 10.1242/dmm.009274 22773757 PMC3484876

[56] Zhou XP, Woodford-Richens K, Lehtonen R, Kurose K, Aldred M, Hampel H, et al (2001) Germline mutations in BMPR1A/ALK3 cause a subset of cases of juvenile polyposis syndrome and of Cowden and Bannayan-Riley-Ruvalcaba syndromes. Am J Hum Genet 69: 704–711. 11536076 10.1086/323703 PMC1226057

[57] Alliman S, Coppinger J, Marcadier J, Thiese H, Brock P, Shafer S, et al (2010) Clinical and molecular characterization of individuals with recurrent genomic disorder at 10q22.3q23.2. Clin Genet 78: 162–168. 10.1111/j.1399-0004.2010.01373.x 20345475

[58] Wuyts W, Reardon W, Preis S, Homfray T, Rasore-Quartino A, Christians H, et al (2000) Identification of mutations in the MSX2 homeobox gene in families affected with foramina parietalia permagna. Hum Mol Genet 9: 1251–1255. 10767351 10.1093/hmg/9.8.1251

[59] Kress W, Schropp C, Lieb G, Petersen B, Busse-Ratzka M, Kunz J, et al (2006) Saethre-Chotzen syndrome caused by TWIST 1 gene mutations: functional differentiation from Muenke coronal synostosis syndrome. Eur J Hum Genet 14: 39–48. 16251895 10.1038/sj.ejhg.5201507

[60] Gillespie JR, Bush JR, Bell GI, Aubrey LA, Dupuis H, Ferron M, et al (2013) GSK-3beta function in bone regulates skeletal development, whole-body metabolism, and male life span. Endocrinology 154: 3702–3718. 10.1210/en.2013-1155 23904355 PMC5053811

